# Supplementary material for: Exploring Healthcare Workers’ Knowledge and Perspectives on Behavioral Risk Factors Contributing to Non-Communicable Diseases: A Qualitative Study in Bushbuckridge, Ehlanzeni District, Mpumalanga Province, South Africa
Source: Int J Environ Res Public Health. 2025 Feb 26;22(3):343. doi: 10.3390/ijerph22030343 (PMC11942398; doi:10.3390/ijerph22030343)
Supplement: Supplementary file 1 [file ijerph-22-00343-s001.zip › ijerph-3332198-File S1.pdf]

Semi-structured interview for health care providers (nurses)

1. Disease burden in the region/district/area

a. What are the most common chronic diseases in this area?
b. Who are the most affected?
c. What are behavioural risk factors responsible for the disease burden in this area?

2. Interventions/programmes in place

a. How are Hypertensive and Diabetes patients managed in your health facility?
b. How are health programmes in your health facility integrated with other stakeholders? E.g, CHCW's, NGO's, etc.
c. What health programmes are there in this community in relation to preventions, control of Behavioural risk factors for Hypertension and Diabetes?
d. What is your facility doing to ensure that patients have information regarding of Hypertension and Diabetes daily.(**Probe screening, Health talks, how often etc)**
e. Which programmes did you put in place to make patients aware of behavioural risk factors for Hypertension and Diabetes?
f. What trainings have you attended in relation to non-communicable diseases prevention and management? What was covered in the trainings?

3. Community awareness of health problems in relation to **behavioural risk factors for NCD's**

a. What is your view of the community's use of the services in place?
b. What are the difficulties?
c. How are the people in this community encouraged to take part in health service delivery?
d. How does your community get information on health? **(Probe on media, community meetings, etc.)**

4. Community participation in health service delivery in relation to **behavioural risk factors for NCD's**

a. How are communities involved in health service delivery?

b. How the community members are organised to participate in health service delivery? **(Probe whether they are organised in committees, groups, etc).**

c. What challenges do you encounter in support the community's participation in health care delivery?

d. What is your view about the level of community participation?

5. Readiness (willingness and ability/capacity) of the communities to engage in health service delivery in relation to **behavioural risk factors for NCD's**

a. How willing and ready are the people in this community to get involved in health service delivery? **(Please, give some examples).**

b. What training has been undertaken for community members to increase their participation in health activities?

6. How can communities be effectively involved in **behavioural risk factors for NCD's?**

a. What would you consider to be the roles of the communities in health care delivery **(Probe for awareness creation, financing, supporting community health worker, etc)**
